# Analysis of community connectivity in spatial transcriptomics data

**DOI:** 10.3389/fams.2024.1403901

**Published:** 2024-07-11

**Authors:** Juan Xie, Kyeong Joo Jung, Carter Allen, Yuzhou Chang, Subhadeep Paul, Zihai Li, Qin Ma, Dongjun Chung

**Affiliations:** 1 The Interdisciplinary Ph.D. Program in Biostatistics, The Ohio State University, Columbus, OH, United States,; 2 Department of Biomedical Informatics, The Ohio State University, Columbus, OH, United States,; 3 Pelotonia Institute for Immuno-Oncology, The James Comprehensive Cancer Center, The Ohio State University, Columbus, OH, United States,; 4 Department of Computer Science and Engineering, The Ohio State University, Columbus, OH, United States,; 5 Global Statistical Sciences, Eli Lilly and Company, Indianapolis, IN, United States,; 6 Department of Statistics, The Ohio State University, Columbus, OH, United States

**Keywords:** spatial transcriptomics, analysis of community connectivity, stochastic block model, Bayesian models, network analysis

## Abstract

**Introduction:**

The advent of high throughput spatial transcriptomics (HST) has allowed for unprecedented characterization of spatially distinct cell communities within a tissue sample. While a wide range of computational tools exist for detecting cell communities in HST data, none allow for the characterization of community connectivity, i.e., the relative similarity of cells within and between found communities—an analysis task that can elucidate cellular dynamics in important settings such as the tumor microenvironment.

**Methods:**

To address this gap, we introduce the analysis of community connectivity (ACC), which facilitates understanding of the relative similarity of cells within and between communities. We develop a Bayesian multi-layer network model called BANYAN for the integration of spatial and gene expression information to achieve ACC.

**Results:**

We demonstrate BANYAN’s ability to recover community connectivity structure via a simulation study based on real sagittal mouse brain HST data. Next, we use BANYAN to implement ACC across a wide range of real data scenarios, including 10× Visium data of melanoma brain metastases and invasive ductal carcinoma, and NanoString CosMx data of human-small-cell lung cancer, each of which reveals distinct cliques of interacting cell sub-populations. An R package banyan is available at https://github.com/dongjunchung/banyan.

## 1 Introduction

The advent of spatial transcriptomics has allowed for unprecedented characterization of tissue architecture in terms of spatially resolved transcript abundance [1]. In particular, *high throughput spatial transcriptomics* (HST) technologies, such as the 10× Visium platform, have become popular due to their deeper transcriptome-wide sequencing depth. The proliferation of HST data has led to the development of several computational tools for discerning cell sub-populations in HST data while considering both gene expression and spatial information. The existing tools span a range of methodological categories, including neural networks [2–4], graph clustering algorithms [5, 6], and Bayesian statistical models [7, 8].

These methods are fundamentally limited in that they do not explicitly model the interactive nature of cell sub-populations in a tissue sample [9]. In other words, the sub-populations derived from existing methods are considered static, and no information is provided on how they relate to one another. Meanwhile, it is known that communication within and between groups of cells is a fundamental driver of healthy and diseased processes in complex tissue [10]. Moreover, Canozo et al. [4] report substantial heterogeneity within traditional mouse olfactory bulb layer annotations, driven in part by spatial variation in intercellular communication patterns. However, detecting higher resolution cell sup-populations with existing tools is challenging as there is a lack of model-based methodology for determining which cell suppopulations may be members of a common broader phenotype (e.g., immune or cancer cell sub-types) based on similar yet distinct gene expression or spatial location patterns. As a consequence, current tools cannot be used to study the *community connectivity structure* of cell sub-populations, i.e., the relative similarity among cells within and between sub-populations.

By studying community connectivity structure, we may obtain valuable insights into the interactive dynamics and spatial heterogeneity of cell sup-populations in challenging settings such as the tumor microenvironment. For example, instead of simply labeling categories of immune cells and cancer cells in a tumor, we may quantify how these important cell sub-populations relate to one another, and how tertiary intermediate sub-populations may be mediating important dynamics within the tumor microenvironment. Furthermore, characterizing community connectivity structure may help inform more biologically informative annotations of ambiguous sub-populations by relating them to more clearly defined sub-populations. Doing so may allow for a more holistic interpretation of all HST cell clusters in the common case when only a few cell clusters correspond clearly to a known cell type.

To address these gaps, we propose BANYAN (**B**ayesian **AN**alysis of communit**Y** connectivity in sp**A**tial single-cell **N**etworks): a Bayesian statistical network model capable of discerning community connectivity structure in HST data. BANYAN draws inspiration from the vast field of biological network analysis [11], and is built on the supposition that HST data is most accurately represented as similarity networks that reflect similarity between cell spots in terms of spatial location and transcriptional profiles. As opposed to simple comparisons of marker gene expression across cell sub-populations, quantification of similarity metrics can more effectively represent the information contained in thousands of gene markers. To this end, BANYAN introduces a Bayesian multi-layer stochastic block model [12, 13] that infers a community connectivity structure to characterize the relationships between cell spots both within and between sub-populations, based jointly on transcriptional and spatial similarity between cell spots. We offer convenient implementation and interactive visualization functionality via the R package BANYAN.

## 2 Methods

**FIGURE 1:**
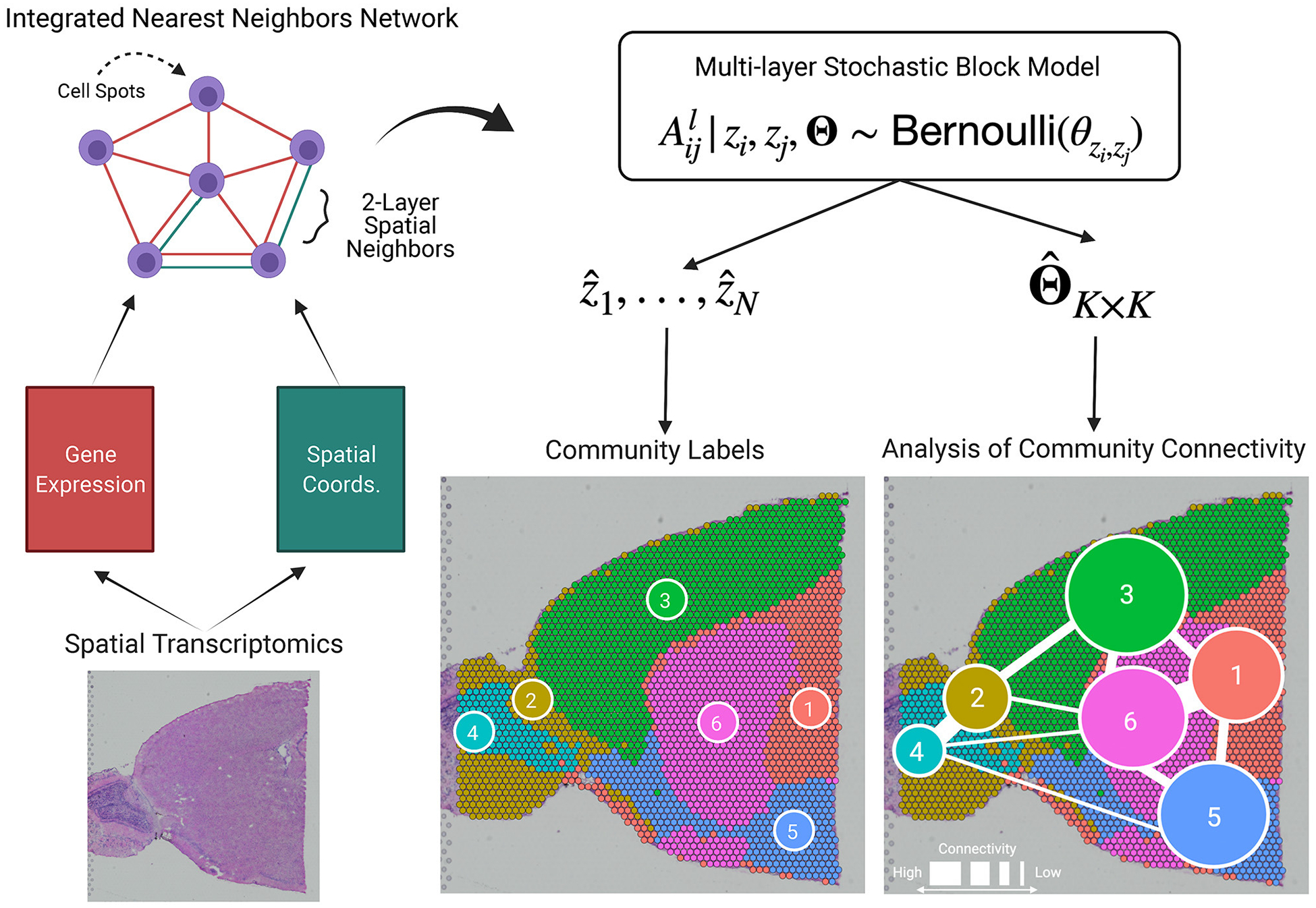
BANYAN introduces the analysis of community connectivity. Spatial transcriptomics platforms yield gene expression and spatial coordinate matrices, which may be used to construct spot-spot nearest neighbor networks that describe how cell spots are similar in terms of gene expression or spatial information. These network structures are passed to a multi-layer stochastic block model (MLSBM). Analysis of community connectivity is achieved through estimation of MLSBM connectivity parameters ${\overset{ˆ}{\Theta }}_{K\times K}$.

BANYAN is the first HST computational tool to allow for *analysis of community connectivity* (ACC), i.e., the process of inferring the similarity of cell spots within and between sub-populations. A graphical representation is given in Figure 1, and the workflow to achieve ACC can be summarized as follows. First, given cell spot-level gene expression features and spatial coordinate data from HST platforms, we construct two spot-spot nearest neighbor networks. These networks are then integrated into a multi-layer graph data structure. Then, we fit a Bayesian multilayer stochastic block model (MLSBM), which assumes that spatial location and gene expression patterns of cell spots arise from a common community structure. The estimated parameters from this model allow us to infer the community structure of the tissue sample by quantifying the relative similarity between cell spots within and between sub-populations.

### 2.1 Data pre-processing

To represent the interactive nature of cells and cell types, we adopt two cell-cell similarity networks as our primary data objects: one for gene expression and another for spatial location. To form the cell spot-cell spot gene expression similarity network, we first apply standard pre-processing steps including scaling, removal of technical artifacts, and identification of highly variable genes [14–16]. We then embed each of the N total cell spots in a lower-dimensional space using principal components analysis (PCA) applied to the top 2,000 most variable genes. To form the cell spot-cell spot gene expression similarity matrix, we represent each cell spot as a node and connect each cell spot to its R closest neighboring cell spots in the gene expression principal component space using a binary edge. We utilize the same approach to construct the spatial cell spot-cell spot similarity network, where principal components are replaced with 2-dimensional spatial coordinates. The resultant data structure is two networks with N nodes, each of degree R. By default, we adopt the widely used heuristic of choosing R as the closest odd integer to $\sqrt{N}$ [17], which allows the number of neighboring spots to increase as the size of the tissue sample increases. With the typical HST experiment yielding a total number of cell spots between 2,000 and 3,000, this heuristic leads to consideration of between third- and fourth-order neighborhood structures (Supplementary Figure S1). Overall, we view R as a tuning parameter that may be adjusted depending on the amount of information sharing desired across a tissue sample.

### 2.2 Model

We develop the core statistical model within BANYAN as an extension of the widely used stochastic block model [18], a flexible generative model for network data that allows for the assessment of community structure based on the frequency of binary edges among and between subsets of nodes. We define $A^{1}$ as the N×N binary adjacency matrix encoding the gene expression similarity network, and $A^{2}$ as the binary adjacency matrix encoding the spatial similarity network. The matrix elements $A_{ij}^{1}$ and $A_{ij}^{2}$ indicate the presence or absence of a binary un-directed edge between nodes i and j for gene expression and spatial information, respectively. We define $𝒜=\left\{A^{1},A^{2}\right\}$ as the multi-layer graph that encodes similarity between cell spots in terms of both gene expression and spatial information. While we focus on the integration of spatial and gene expression information, our proposed framework may be extended to L layers to incorporate other sources of information from multiplexed experimental assays.

Given the multi-layer graph data 𝒜, we assume that the absence or presence of edges in each layer between each pair of nodes i and j follows a Bernoulli distribution with probability of an edge $\theta_{z_{i},z_{j}}$, where $z_{i}\in \{1,\ldots ,K\}$ denotes the latent cell spot sub-population assignment for cell spot i. We refer to such a model as MLSBM. Formally, we assume for l=1,2,

(1) $$A_{ij}^{l}|z,\Theta \overset{ind}{\sim }\text{Bernoulli}\left(\theta_{z_{i},z_{j}}\right)\;\text{for}\;i<j=1,\ldots ,N,$$

where $z=\left(z_{1},\ldots ,z_{N}\right)$, and Θ is a K×K *connectivity matrix* with diagonal elements $\theta_{rs}$ for r=s=1,…,K controlling the probability of an edge occurring between two cell spots in the same sub-population, and off-diagonal elements $\theta_{rs}$ for r<s=1,…,K controlling the probability of an edge occurring between two nodes in different cell spot sub-populations. Importantly, Model (1) implies that connections among cell spots in the gene expression and spatial layers are governed by a common set of community structure parameters z and Θ. Note that in the graph 𝒜, the edges corresponding to the cases that $A_{ij}^{1}=1$ and $A_{ij}^{2}=1$ usually constitute the core of the community, while the edges corresponding to the cases that $A_{ij}^{1}=1$ and $A_{ij}^{2}=0$, or $A_{ij}^{1}=0$ and $A_{ij}^{2}=1$, usually constitute the outskirts of the community. Given Model (1) and data 𝒜, our primary inferential objective is to characterize the cell spot-cell spot interaction both *within* and *between* them by estimating the parameters Θ, which we accomplish using a Bayesian approach as described below.

### 2.3 Bayesian inference

#### 2.3.1 Priors

To achieve a fully Bayesian parameter estimation scheme, we assign prior distributions to all model parameters. We adopt available conjugate priors to obtain closed-form full conditional distributions of all model parameters, allowing for straightforward Gibbs sampling. For the latent cell sub-population indicators $z_{1},\ldots ,z_{N}$, we assume a conjugate multinomial-Dirichlet prior with $z_{i}\overset{iid}{\sim }\text{Categorical}(\pi )$ for i=1,…,N, and $\pi \sim \text{Dirichlet}\left(\alpha_{1},\ldots ,\alpha_{K}\right)$, where $\pi =\left(\pi_{1},\ldots ,\pi_{K}\right)$ controls the relative size of each cell sub-population to allow for a heterogeneous distribution of cell type abundances. We adopt a conjugate Beta-Bernoulli prior for Θ by assuming $\theta_{rs}\overset{iid}{\sim }\text{Beta}\left(\beta_{1},\beta_{2}\right)$ for r<s=1,…,K. As a default, we opt for weakly informative priors by setting $\alpha_{1}=\alpha_{2}=\ldots =\alpha_{K}=1$ and $\beta_{1}=\beta_{2}=1$ [19].

#### 2.3.2 Markov chain Monte Carlo (MCMC) algorithm

The model proposed in Sections 2.2 and 2.3.1 allows for closed-form full conditional distributions of all model parameters. Thus, we adopt the following Gibbs sampling algorithm for parameter estimation. In practice, we recommend initializing the indicators $z_{i},\ldots ,z_{N}$ using a heuristic graph clustering method such as the Louvain algorithm [20] applied to $A^{1}$ to facilitate timely model convergence.

1. Update π from its full conditional $(\pi |A,z,\Theta )\sim \text{Dirichlet}\left(a_{1},\ldots ,a_{K}\right)$, where $a_{k}=\alpha_{k}+n_{k}$, and $n_{k}$ is the number of nodes assigned to cell sub-population k at the current MCMC iteration, i.e., $n_{k}=\sum_{i=1}^{N}I_{z_{i}=k}$.
2. For r≤s=1,…,K, update $\theta_{rs}$ from
$$\left(\theta_{rs}|A,z,\pi \right)\sim \text{Beta}\left(\beta_{1}+A[rs],\beta_{2}+n_{rs}-A[rs]\right)$$
  where A[rs] are the number of observed edges between communities r and s across both layers, and $n_{rs}=2\left(n_{r}n_{s}-\right.n_{r}I(r=s))$ are the number of possible edges between communities r and $s,n_{r}$ is the number of nodes assigned to cell sup-population r, and I(r=s) is the indicator function equal to 1 if r=s and 0 otherwise.
3. For i=1,…,N, update $z_{i}$ from $\left(z_{i}|z_{-i},A,\pi ,\Theta \right)\sim \text{Categorical}\left(\rho_{i}\right)$, where $\rho_{i}=\left(\rho_{i1},\ldots ,\rho_{iK}\right)$ and
$$\rho_{ik}=\pi_{k}\left(\overset{2}{\underset{l=1}{\prod }}\underset{j\neq i}{\prod }\theta_{k,z_{j}}^{A_{ij}^{l}}{\left(1-\theta_{k,z_{j}}\right)}^{1-A_{ij}^{l}}\right)\;\left(\overset{2}{\underset{l=1}{\prod }}\underset{h\neq i}{\prod }\theta_{z_{h,k}}^{A_{hi}^{l}}{\left(1-\theta_{z_{h},k}\right)}^{1-A_{hi}^{l}}\right)$$

#### 2.3.3 Label switching

Label switching is a ubiquitous issue faced by models whose likelihood is invariant to permutations of a latent categorical variable such as z. Consequently, stochastically equivalent permutations of z may occur over the course of MCMC sampling, causing the estimates of all community-specific parameters to be conflated, thereby jeopardizing the accuracy of model parameter estimates. Previous approaches for addressing label switching rely on re-shuffling posterior samples after completion of the MCMC algorithm [21]. However, such methods rely on prediction and are subject to prediction error. To protect against label switching within the MCMC sampler, we adopt the canonical projection of z proposed by Peng and Carvalho [22], who restrict updates of z to the reduced sample space 𝒵={z:ord(z)=(1,…,K)}, wherein label switching is less likely due to the restricted sample space. In practice, we manually permute z at each MCMC iteration such that community 1 appears first in z, community 2 appears second in z, *et cetera*. Finally, we estimate z using the maximum *a posteriori* (MAP) estimate across all post-burn MCMC samples [19].

### 2.4 Analysis of community connectivity

Estimation of the MLSBM model parameters Θ with the corresponding maximum *a posteriori* estimates $\overset{ˆ}{\Theta }$ allows for inference of community connectivity structure in HST data. While the estimated community labeling vector $\overset{ˆ}{z}$ is what we use to define communities, the elements of $\overset{ˆ}{\Theta }$ describe how cell spots within and between communities relate to one another, thereby characterizing community connectivity. Specifically, elements ${\overset{ˆ}{\theta }}_{rs}$ reflect the estimated probability of a randomly chosen cell spot in community r sharing a nearest neighbors edge in 𝒜 with a cell spot in community s. When $r=s,{\overset{ˆ}{\theta }}_{rs}$ reflects the average connectivity within a community, which may be used to assess the relative homogeneity of a community. Heterogeneous communities tend to have lower average within-community connectivity, while more homogeneous communities tend to have higher within-community connectivity. Likewise, when $r\neq s,{\overset{ˆ}{\theta }}_{rs}$ represents the probability of connection between cell spots in two distinct communities. This between-community connectivity measurement allows us to discern closely related communities that may contain similar cell types from more distinct communities. Taken together, these between and within-community connectivity parameters capacitate analysis of community connectivity.

### 2.5 Software implementation

We provide the R package banyan for convenient implementation of the proposed workflow. The banyan package efficiently implements Bayesian estimation using custom Gibbs sampling algorithms implemented in C++ using Rcpp. The core model fitting functions integrate seamlessly with standard Seurat [23] data structures, allowing users to easily incorporate ACC into existing HST analysis workflows. Further, banyan allows users to investigate community connectivity using external sub-population labels, thereby encouraging widespread utility of ACC. As clustering algorithms for HST proliferate—each with different assumptions and optimal use cases, utilizing BANYAN for *post-hoc* community connectivity analysis will help elucidate tissue heterogeneity across the widest possible range of application settings. We developed both interactive and static visualization functions for interrogation of BANYAN sub-population labels and community connectivity structure. The banyan package interfaces seamlessly with standard Seurat workflows, and is freely available at https://github.com/dongjunchung/banyan.

## 3 Result

### 3.1 Simulation studies show BANYAN effectively identifies underlying community connectivity structures under a broad range of signal-to-noise ratio settings

We designed a simulation study to validate the performance of the MLSBM employed by BANYAN. We adopted a publicly available sagittal mouse brain data set [24] sequenced with the 10× Visium platform. In our simulation data, we manually allocated the N=2,696 total cell spots in the original sagital mouse brain data set into five spatially contiguous mouse brain layers. Assuming that a community structure is given as

$$\Theta =\left[\begin{array}{ccccc} \theta & 0.1 & 0.1 & 0.1 & 0.1\\ 0.1 & \theta & 0.1 & 0.1 & 0.1\\ 0.1 & 0.1 & \theta & 0.1 & 0.1\\ 0.1 & 0.1 & 0.1 & \theta & 0.1\\ 0.1 & 0.1 & 0.1 & 0.1 & \theta \end{array}\right],$$

we defined the *signal-to-noise ratio* (SNR) of the simulated gene expression network as SNR=θ/0.1, i.e., the ratio of the within- to between-community connectivity. SNR values much greater than 1 give rise to a strong community structure in the simulated data, while SNR values close to 1 result in a weaker community structure. We do not consider values of SNR below 1, as the resultant disassortative community structure is not reflective of cell type structure in HST data. In addition, we note that SNR was close to 10 in all the real data applications we consider in the following sections, i.e., the ranges of SNR we considered in these simulation studies are significantly lower than those we observe in real datasets. Hence, given the usual SNR levels we observe in real datasets, BANYAN is expected to effectively recover the underlying community connectivity structure.

**FIGURE 2:**
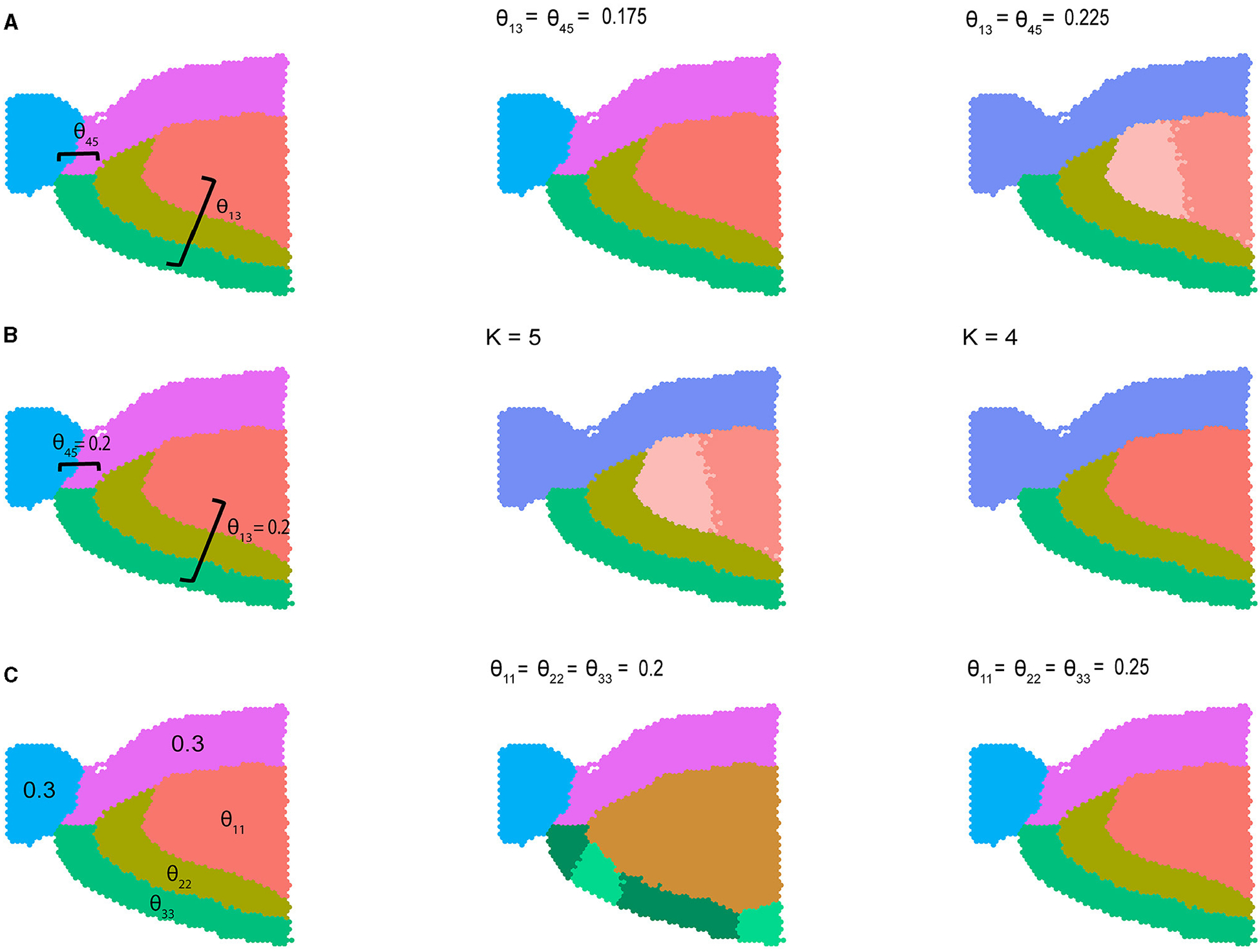
Simulation studies using sagittal mouse brain tissue sample with five cell spot clusters. **(A)** Simulation setting 1, where within-community connectivity (WCC) is 0.3, two between-community connectivity (BCC) ($\theta_{12}$ and $\theta_{45}$) vary from 0.175 to 0.225, and the rest BCC are set to 0.1. **(B)** Simulation setting 2, where WCC is 0.3, two BCC $\left(\theta_{13}\right.$ and $\left.\theta_{45}\right)$ were set to 0.2, the rest BCC were set to 0.1. The number of communities varies from 5 to 4. **(C)** Simulation setting 3, where all the BCC were set to 0.1, two WCC were set to 0.3, and we vary three WCC from 0.2 to 0.25.

We explore the effects of varying between-community connectivity and within-community connectivity to modulate the signals-to-noise ratio (SNR). We used the general community structure given by

(2) $$\Theta =\left[\begin{array}{lllll} \theta_{11} & \theta_{12} & \theta_{13} & \theta_{14} & \theta_{15}\\ \theta_{21} & \theta_{22} & \theta_{23} & \theta_{24} & \theta_{25}\\ \theta_{31} & \theta_{32} & \theta_{33} & \theta_{34} & \theta_{35}\\ \theta_{41} & \theta_{42} & \theta_{43} & \theta_{44} & \theta_{45}\\ \theta_{51} & \theta_{52} & \theta_{53} & \theta_{54} & \theta_{55} \end{array}\right],$$

where by default $\theta_{ij}=0.1$ for i≠j and $\theta_{ij}=0.3$ otherwise. In Figure 2 we visualize results from three different variations of Equation (2) described above. In the first setting, we varied the between-community connectivity between pair of spatially disjoint communities 1 and 3 and the pair of bordering communities 4 and 5 to analyze the interplay between between-community connectivity and spatial co-localization (Figure 2A). When we selectively decrease SNR by setting $\theta_{13}=\theta_{45}=0.175$, BANYAN is still able to perfectly recover sub-population labels. However, when we further decrease SNR by increasing $\theta_{13}=\theta_{45}=0.225$, we find that sub-population inference is corrupted. Notably, as the SNR approaches 1, BANYAN merges sub-populations 4 and 5 first, as they feature high between-community connectivity *and* spatial proximity. Alternatively, BANYAN splits sub-population 1 into two distinct communities instead of combining sub-populations 1 and 3, which are spatially disjoint. In Supplementary Figure S2, we visualize this trend across a finer grid of $\theta_{13}$ and $\theta_{45}$. In Figure 2B, we demonstrate this phenomenon from different perspective, in which decreasing the clustering resolution from K=5 to K=4 features merging of the two distinct sub-population 1 components due to their spatial proximity and high connectivity. Finally, in setting 3 (Figure 2C), we investigated the effect of selectively decreasing within-community connectivity parameters for sub-populations 1, 2, and 3. We find that at low SNR settings of $\theta_{11}=\theta_{22}=\theta_{33}=0.2$, BANYAN is unable to properly allocate cell spot labels, while increasing the SNR by increasing $\theta_{11}=\theta_{22}=\theta_{33}=0.25$ results in correct recovery of ground truth labels. In Supplementary Figure S3, we provide results from across a finer grid of $\theta_{11},\theta_{22}$, and $\theta_{33}$. The results from settings 1–3 in Figure 2 highlight a characteristic of the spatially-aware MLSBM, namely that the model places a preference on merging spatially neighboring communities instead of spatially separate communities when the SNRs for each pair are equally low (i.e., approaches SNR = 1).

### 3.2 Identifying cellular interplay in human melanoma brain metastases

Brain metastases are a common cancer complication, arising most often from lung cancer, breast cancer, and melanoma and occurring in nearly 30% of patients with solid tumors [25]. In the United States, an estimated 98,000 to 170,000 patients are diagnosed with brain metastases each year, and the incidence is increasing [26]. Due to the fact that conventional therapies can rarely cure brain metastases, researchers have been seeking alternative treatment options, and immunotherapy is one promising candidate [27]. In recent years, many scientific efforts have been devoted to investigating the interaction between the immune system and the tumor microenvironment (TME) of brain metastases, shedding light on the immune biology of brain metastases. For instance, Sudmeier et al. [28] reported that human brain metastases are well infiltrated by $\text{CD}8^{+}$ T cells.

**FIGURE 3:**
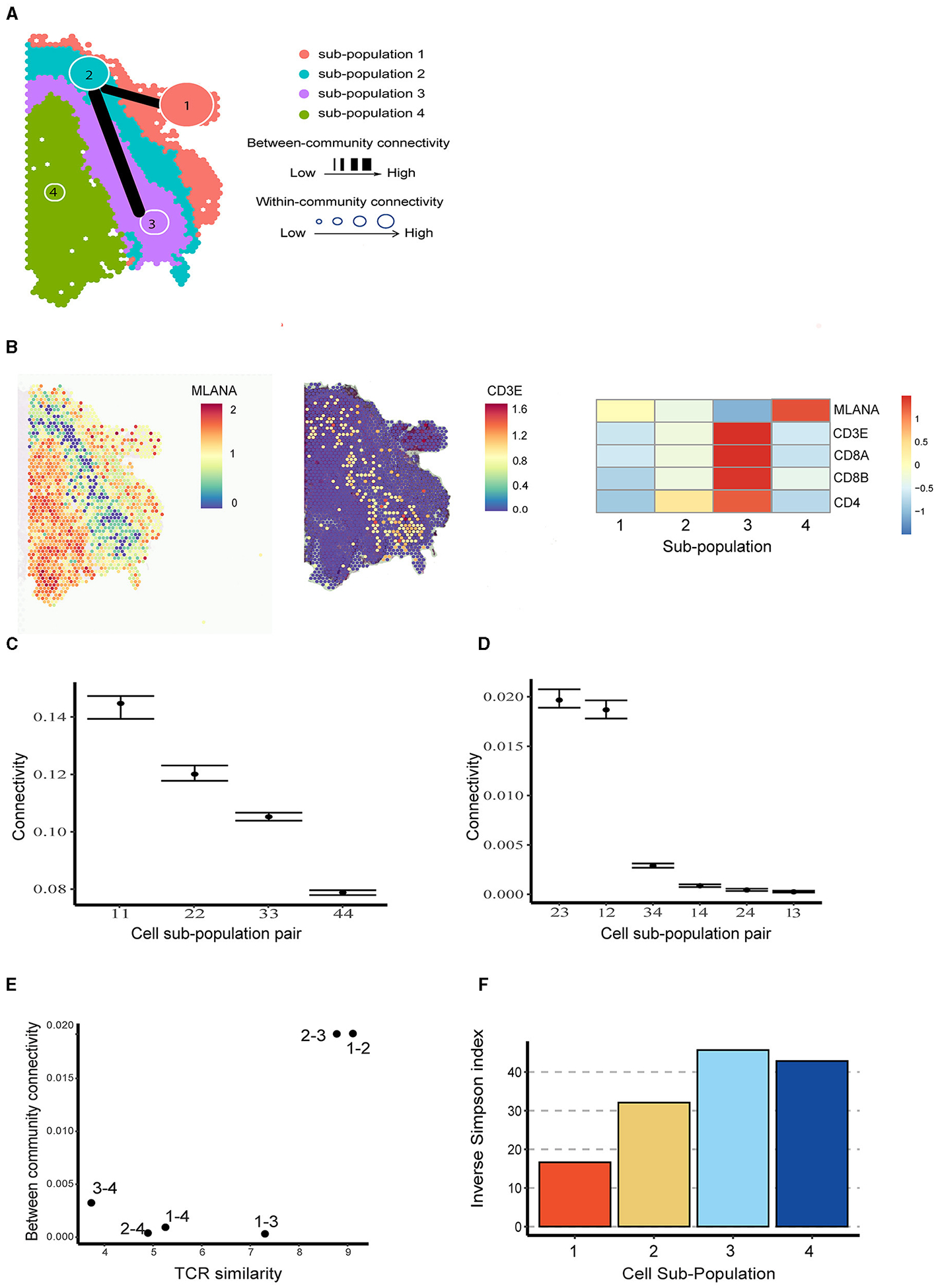
Community structure in melanoma brain metastasis data. **(A)** Inferred cell spot sub-population labels from BANYAN. Line width indicates the between-community connectivity level, where the thicker the line, the higher the connectivity. Node size reflects within-community connectivity level, where the larger the node, the higher the connectivity. **(B)** Spatial plot and heatmap for tumor and T cell markers. **(C)** Within-community connectivity. **(D)** Between-community connectivity intervals. **(E)** Relationships between T cell receptor similarity and between-community connectivity. **(F)** The repertoire diversity for each sub-population.

To better understand the spatial distribution of immune cells in brain metastases TME and their interactive relationship with tumor cells, we applied BANYAN to the human melanoma brain metastasis sample from Sudmeier et al. [28], who applied spatial transcriptomics and identified distinct tumor, inflammatory, and blood cell sub-populations. Using BANYAN, we identified four spatially distinct spot sub-populations (Figure 3A), characterized the function of each sub-population in the TME using known marker genes (Figure 3B; Supplementary Figure S4), and studied the similarity structure among cell spots within and between sub-populations (Figures 3C, D). The identified sub-populations from BANYAN closely resemble the TME regions reported by Sudmeier et al. [28]. BANYAN sub-population 1 corresponds to blood cells, sub-population 2 to inflammatory immune cells, sub-population 3 to tumor-inflammatory adjacent cells, and sub-population 4 to the tumor region, respectively. Functional annotation based on the expression of marker genes (Figure 3B; Supplementary Figure S4) further suggests sub-population 1 consists of naive cells, while both sub-populations 2 and 3 are ${\text{CD}8}^{+}$ T cells, and the expression of ${\text{CD}8}^{+}$ T cell markers are higher in sub-population 3 compared to those in sub-population 2.

We then utilized BANYAN to implement ACC and characterize the interplay among the four identified sub-populations—a unique functionality not offered by other HST analysis tools. When investigating within-community connectivity parameters in Figure 3C (Supplementary Figure S5), we observe a decreasing density of cell-cell connectivity as we move from outside to within the tumor. This pattern suggests additional cellular heterogeneity within the tumor relative to the surrounding inflammatory and blood tissue components. When consulting the between-community connectivity parameters in Figure 3D, we find two distinct pairs of cell sub-populations: (2,3) and (1,2), which feature significantly higher inter-connectivity than all other pairs of sub-populations. These three sub-populations comprise the tumor-external components of the tissue sample and reflect a relatively high degree of inter-connectivity between blood, immune, and tumor-adjacent sub-populations. In comparison, the tumor region (sub-population 4) featured significantly lower inter-connectivity with the rest of the tissue sample, as evidenced by the significantly lower between-connectivity parameter estimates for the pairs of (3,4), (1,4), and (2,4) in Figure 3D.

To better understand connectivity, we further analyzed the paired single-cell T cell receptor (TCR) sequencing data in terms of repertoire overlap and diversity (Supplementary Section S1). It turned out that the pairs with higher BCC values correspond to those with higher TCR similarity (Figure 3E). Furthermore, sub-populations with higher WCC values (e.g., sub-populations 1 and 2) exhibited lower repertoire diversity compared to the ones with lower WCC values (Figure 3F). The above relationships between WCC/BCC and TCR repertoire indicate that ACC holds the potential to uncover cellular dynamics under the setting of TME.

### 3.3 Discovering community structure in invasive ductal carcinoma

Accounting for roughly 25% of all non-dermal cancers in women, breast cancer ranks as the most common non-dermal female-specific cancer type, and narrowly the most common cancer type across both sexes [29]. Of all sub-types, invasive ductal carcinoma (IDC) is the most common and most severe, accounting for roughly 80% of all breast cancers in women [30]. While previous authors have used spatial transcriptomics to study IDC samples relative to ductal carcinoma in situ (DCIS) samples [31], IDC has yet to be studied through the lens of community structure due to the lack of computational tools available for performing ACC with HST data.

**FIGURE 4:**
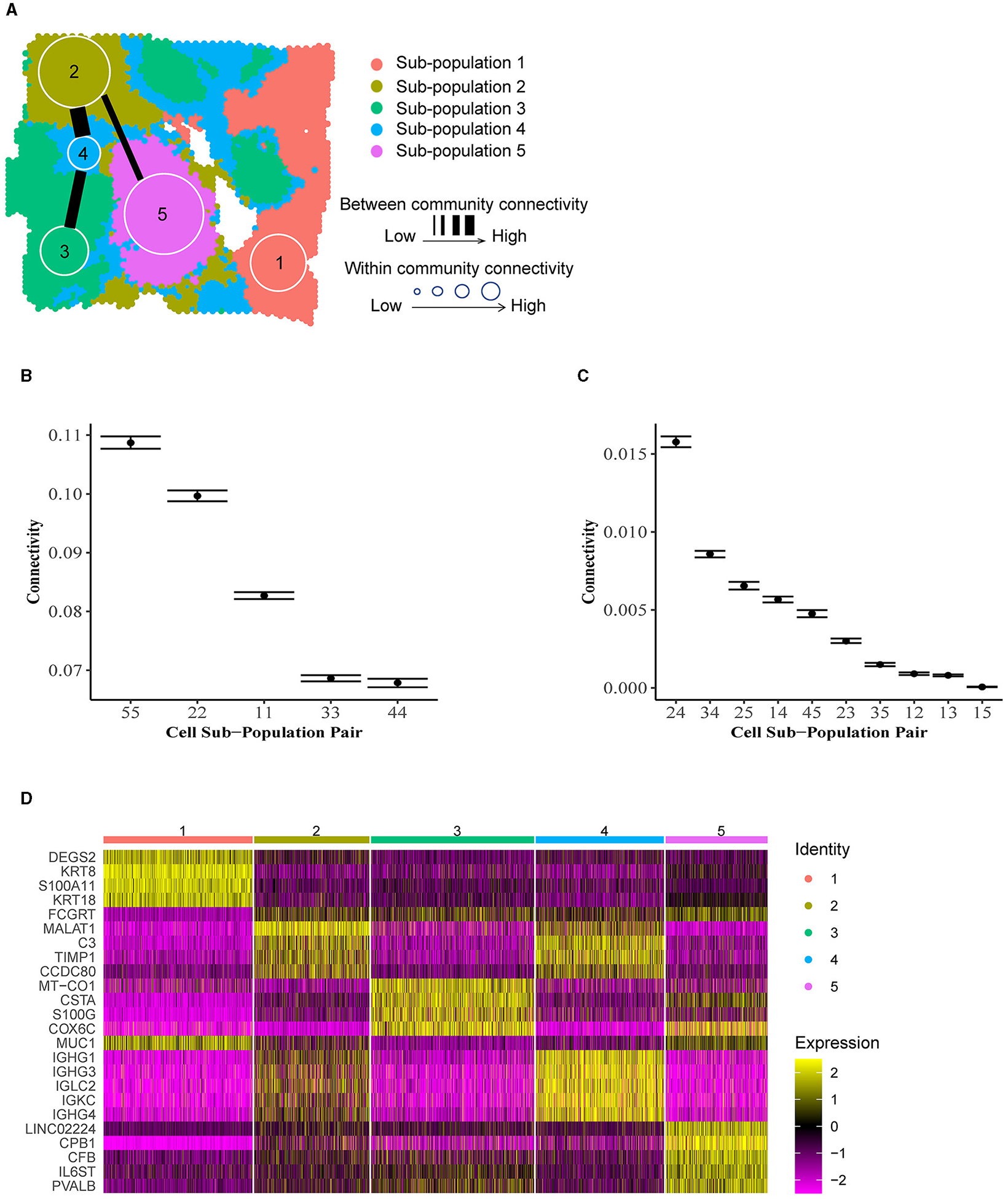
Community structure in invasive ductal carcinoma. **(A)** Inferred cell spot sub-population labels from BANYAN. **(B)** Within-community connectivity parameters reflect the homogeneity of sub-populations. Higher connectivity values reflect higher homogeneity within sub-populations. **(C)** Between-community connectivity parameters reflect the relative similarity of cell spots between sub-populations. Higher connectivity values reflect more similarity between sub-populations. **(D)** Normalized expression of differentially expressed sub-population markers genes.

To illustrate ACC in the tumor microenvironment, we applied BANYAN to a publicly available IDC sample sequenced with the 10× Visium platform [32]. We identified five spatially distinct cell spot sub-populations (Figure 4A), and then identified community structure by computing posterior estimates of within and between-community connectivity parameters, as displayed in Figures 4B, C, respectively. Finally, to interpret each sub-population in terms of IDC biology, we computed the most differentially expressed genes between each sub-population and all others using the Wilcoxon rank-sum test implemented in the Seurat package (details in Supplementary material) (Figure 4D). Figure 4D displays a clear block structure in the expression of sub-population marker genes, indicating a strong community structure signal in the data. When considered together with their spatial distribution (Supplementary Figure S6), these marker genes can be used to obtain many interesting biological insights regarding the community structure of the IDC sample. For instance, the *S100A11* gene, a marker for sub-population 1, is a diagnostic marker in breast cancers [33] and has been implicated in aggressive tumor progression [34]. Further, *KRT8* is used to differentiate aggressive grades of IDCs [35]. While outside of the context of IDCs, *DEGS2* has been shown to play a role in the invasion and metastasis of colorectal cancer [36]. Taken together, these marker genes suggest that sub-population 1 contains a relatively high abundance of aggressive and invasive cancer cell types. On the other hand, sub-population 2 featured marker genes such as *MALAT1* that are associated with tumor suppressive behaviors in IDCs [37]. Another marker gene for sub-population 2, *CCDC80*, has been linked with tumor suppressive functions, albeit not in the context of IDCs [38].

Given these brief characterizations of sub-populations 1 and 2 available from the existing literature, we may hypothesize that these groups of cell spots are in some sense opposed in terms of their role within the tumor based on their transcriptional profiles. Indeed, these sub-populations also reside spatially at opposite ends of the tumor slice. We may investigate the similarity or dissimilarity of these sub-populations 1 and 2 using the between-community connectivity parameters presented in Figure 4C. We find that the estimate of this parameter is near zero [as evidenced by the 95% credible for community pair (1,2) in Figure 4C], supporting our hypothesized dissimilarity between sub-populations 1 and 2. In fact, sub-population 1 featured very low between-community connectivity with all other sub-populations besides sub-population 4 [e.g., significantly higher connectivity was featured between sub-populations pairs (1,4) than (1,2) as shown in Supplementary Figure S7], which occupies a heterogeneous “background” position in the spatial landscape of the tissue sample (Figure 4A) and therefore featured relatively high connectivity with all other communities. This spatial heterogeneity is accompanied by relatively low within-community connectivity (Figure 4B), which indicates that spot-spot similarities are less common between cell spots in sub-population 4 than in other sub-populations. In Figure 4D, it can be seen that many of the marker genes for sub-population 2 are shared by sub-population 4, including *MALAT1*, suggesting a similarity between these two sub-populations in terms of transcriptional profiles. In addition to the marker genes shared with sub-population 2, sub-population 4 features several of its own distinct marker genes, namely the immunoglobulin heavy chain-encoding RNAs *IGHG1* and *IGHG3*. These genes themselves have been shown to feature tumor suppressive tendencies via promotion of B cell-specific immunoglobulin [39], and have been associated with increased patient survival [40]. This observation of functional similarity between sub-populations 2 and 4 is validated by Figure 4C, which clearly shows the highest estimated between-community connectivity in the data occurring between sub-populations 2 and 4. Taken together, these observations may lead us to reason that the sub-population 1 vs. 2 dynamic described previously is linked via the more heterogeneous yet still tumor suppressive-like sub-population 4. While these observations would require further experimental validation to confirm, they showcase the unique ability of BANYAN to describe community structure in the data.

### 3.4 Discovering spatial niches in human non-small cell lung cancer tissues

Next, we applied BANYAN to public data from the NanoString CosMx Spatial Molecular Imager (SMI) platform [41]. The original dataset consists of measurements of eight samples from five non-small-cell lung cancer (NSCLC) formalin-fixed paraffin-embedded (FFPE) tissues, with a total of 800,327 cells. Here we used one of eight samples (lung 5, replicate 1) for the illustration purpose. We further extracted cells corresponding to basal, macrophage, ${\text{CD}4}^{+}$ T, and ${\text{CD}8}^{+}$ T cells types based on the annotation using the Azimuth Healthy Human Lung reference [14, 42]. Finally, we randomly downsampled the original data to 8,000 cells to aid in readability and allow for more rapid MCMC convergence.

**FIGURE 5:**
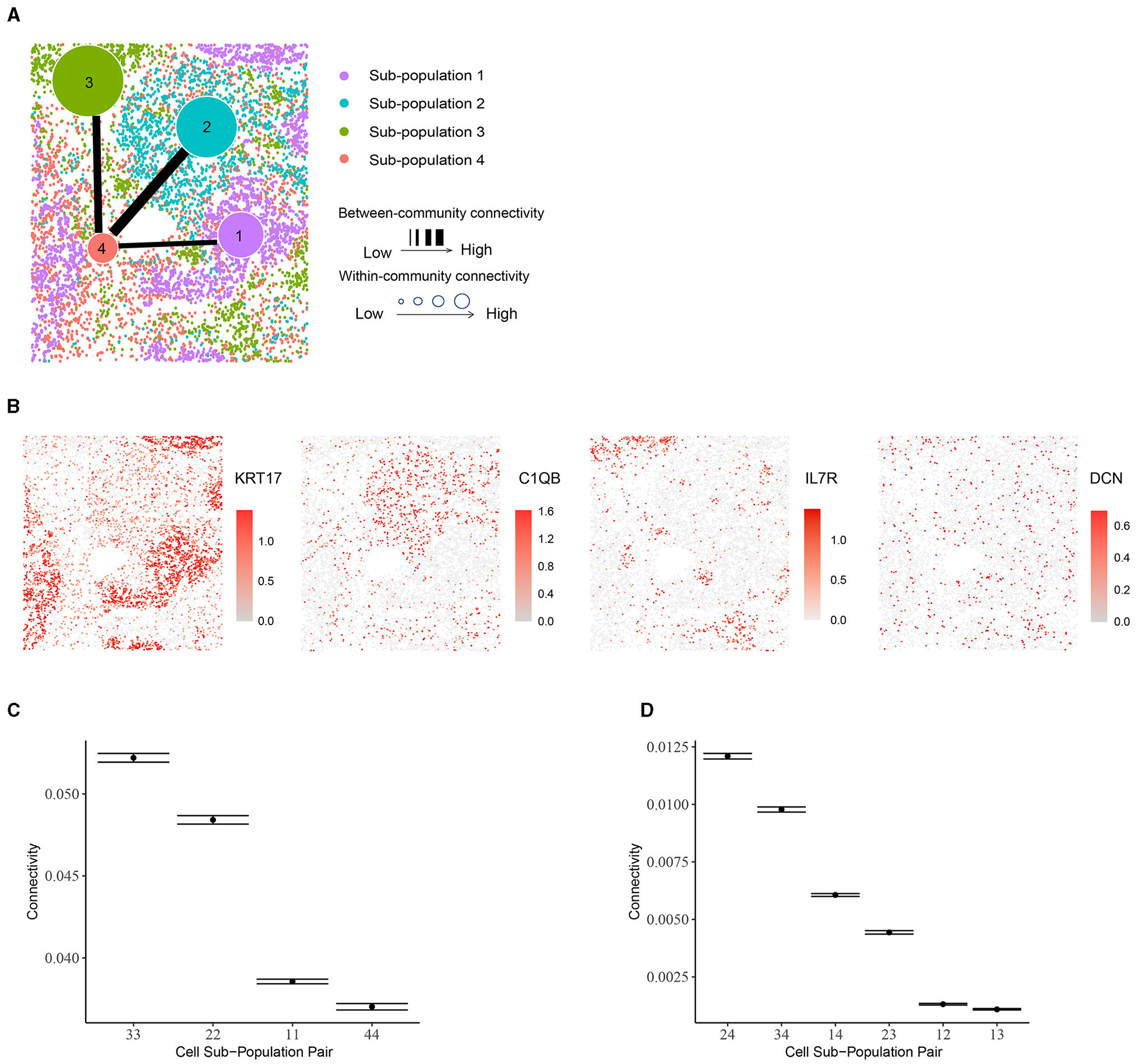
Community structure in human non-small-cell lung cancer tissue. **(A)** Inferred sub-populations from BANYAN. **(B)** Spatial plots for marker genes for each sub-population. **(C)** Within-community connectivity between cells belonging to each sub-population. **(D)** Between-community connectivity between cell sub-populations.

Using BANYAN, we identified four sub-populations (Figure 5A), investigated the marker genes for each sub-population (Figure 5B), and examined the within- and between-community connectivity (Figures 5C, D). The identified four sub-populations closely matched the spatially resolved neighborhood niches reported in the previous literature [43]. Each of BANYAN sub-populations 1 to 4 mainly correspond to tumor cells, myeloid-enriched stroma cells, lymphoid structures, and stroma cells, respectively. The spatial distribution of markers for each sub-population confirmed this correspondence. For instance, *KRT17* is a specific marker for basal cells and commonly used diagnostic marker for tumors [44], and it is highly expressed in sub-population 1. *C1QA*, *C1QB*, and *C1QC* are markers for macrophages [45] (macrophages are myeloid lineage cells [46]), and they are significantly highly expressed in sub-population 2. *IL7R* is a lymphoid-associated gene and we observed its over-expression in sub-population 3.

While examining these sub-populations, we found that the sub-population 4 (stroma cells) is more heterogeneous than the other sub-populations. It consists of all four cell types and each cell type constitutes a fair proportion of the sub-population, while other sub-populations are mostly dominated by only one cell type. This heterogeneity differences among sub-populations may further explain the within-community connectivity: sub-population 3 is dominated by ${\text{CD}4}^{+}$ and ${\text{CD}8}^{+}$ T cells (proportion around 90%) and has the highest within-community connectivity. Likewise, nearly 81% of sub-population 2 are macrophages, potentially explaining its second-highest within-community connectivity. For sub-population 1, 99% of the cells are tumor cells, which themselves display high heterogeneity. In the case of between-community connectivity, as before, the observations may be mainly explained by spatial adjacency: sub-population 4 neighbors with all the other sub-populations and the connectivity involved in this sub-population are high [e.g., the (2,4), (3,4) and (1,4) pairs].

## 4 Discussion

We have proposed BANYAN: a network-based statistical framework for the analysis of community connectivity in HST data. In our simulation study, we validate BANYAN’s ability to recover the community connectivity structure, even in the case of relatively low SNR, by considering both gene expression similarity and spatial proximity. We applied BANYAN to human melanoma brain metastases, human breast cancer, and human lung cancer, to illustrate its utility in applied settings. In the human melanoma brain metastasis case study, within-community connectivity parameters indicated increasing within-community heterogeneity as we move from outside to within the tumor. In addition, between-community connectivity parameters indicated a higher degree of inter-connectivity between blood, immune, and tumor-adjacent subpopulations, compared to those associated with the tumor region. Besides, we found interesting relationships between community WCC/BCC and TCR repertoire, which indicates that ACC holds the potential to uncover cellular dynamics under the setting of TME. In the breast cancer case study, we found a strong community structure, with sub-populations marked by both invasive cancer and cancer-suppressive marker genes. Using community structure parameters, we also identified an intermediate sub-population between these two. In the human-small-cell lung cancer case study, we observe the relevance of within-community connectivity with the heterogeneity of each cell spot cluster, as illustrated with the stroma cell sub-population.

There are several ways our work may be extended. First, often the SBM is refined to accommodate heterogeneous degree distributions among nodes, i.e., *degree correction* [47]. By making this methodological extension to the MLSBM at the core of BANYAN, one could relax our assumption that each cell spot features the same number of neighbors and thereby allow for certain cells spots to feature more connections to the rest of the tissue than other cell spots, such as those on the periphery of the tissue sample. Learning the degree of each cell spot would then inform the detection of highly connected “hub” regions, or weakly connected “satellite” regions of a tissue sample. Second, it is possible to allow gene expression and spatial information to be weighed in a data-adaptive manner, although tuning of appropriate weights would be necessary. Third, another extension could be to relax the assumption that gene expression and spatial location layers are governed by common community structure parameters, and instead allow for layer-specific interpretations of community structure. Fourth, the inherent complexity of network data structures leads to a heavy computational burden for large HST experiments. While we implement our proposed MCMC sampling algorithm using efficient Rcpp routines, BANYAN still requires significantly more computational time than non-network statistical methods [7, 8]. Further optimization would help to reduce the computational burden of community connectivity analysis. Finally, while BANYAN provides the first statistical framework for quantifying community connectivity structure in HST data, further extensions could be made to link BANYAN with methods for predicting cell-cell interactions using data such as ligand-receptor pair status of cells. By doing so, one could refine the general notion of cell spot connectivity to cell spot interaction, which is of major interest in HST data analysis. In this sense, BANYAN establishes a promising statistical framework that may be extended to a wide range of analyses focused on investigating the interactive nature of HST data.

## Supplementary Material

Data Sheet 1

## Data Availability

Publicly available datasets were analyzed in this study. This data can be found here: human melanoma brain metastasis data (https://www.ncbi.nlm.nih.gov/geo/query/acc.cgi?acc=GSE179572), invasive ductal carcinoma data (https://support.10xgenomics.com/spatial-geneexpression/datasets/1.0.0/V1_Breast_Cancer_Block_A_Section_1), and human non-small cell lung cancer CosMx data (https://nanostring.com/products/cosmx-spatialmolecular-imager/nsclc-ffpe-dataset/). The proposed approach was implemented as an open-source R package “banyan” and it is publicly available at https://github.com/dongjunchung/banyan.
