# Supplementary material for: Analysis of community connectivity in spatial transcriptomics data
Source: Front Appl Math Stat. Author manuscript; Available in PMC 2025 Jun 5. (PMC12140621; doi:10.3389/fams.2024.1403901)
Supplement: Data Sheet 1 [file NIHMS2084991-supplement-Data_Sheet_1.pdf]

frontiers

Supplementary Material

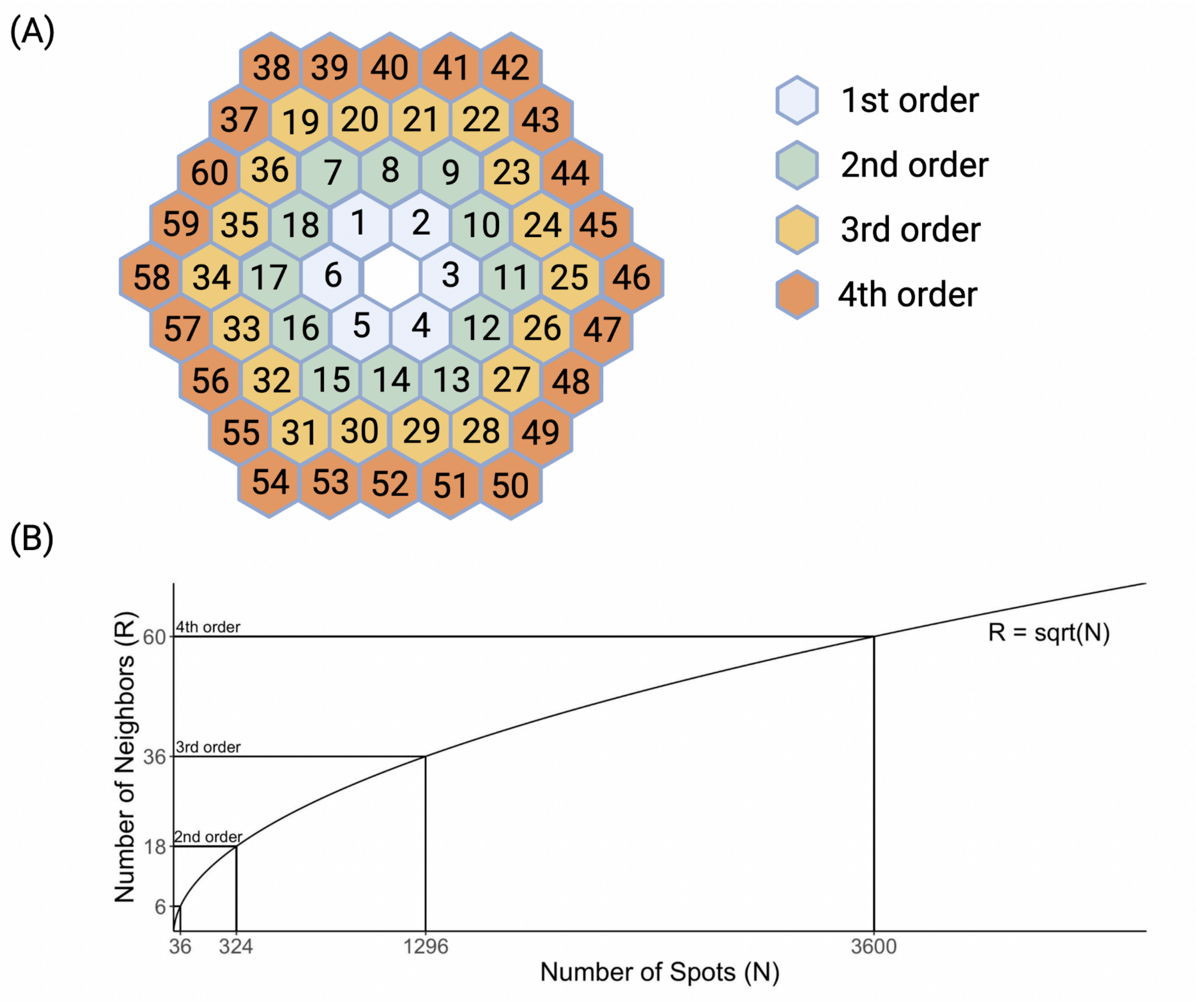

Figure S1. Graphical depiction of the relationship between the number of neighbors and the neighbor order. (A) Hexagonal neighborhood structure for an interior cell spot shown with 1st through 4th order neighbors. (B) Suggested relationship between the number of cell spots (N) and the number of nearest neighbors (R).

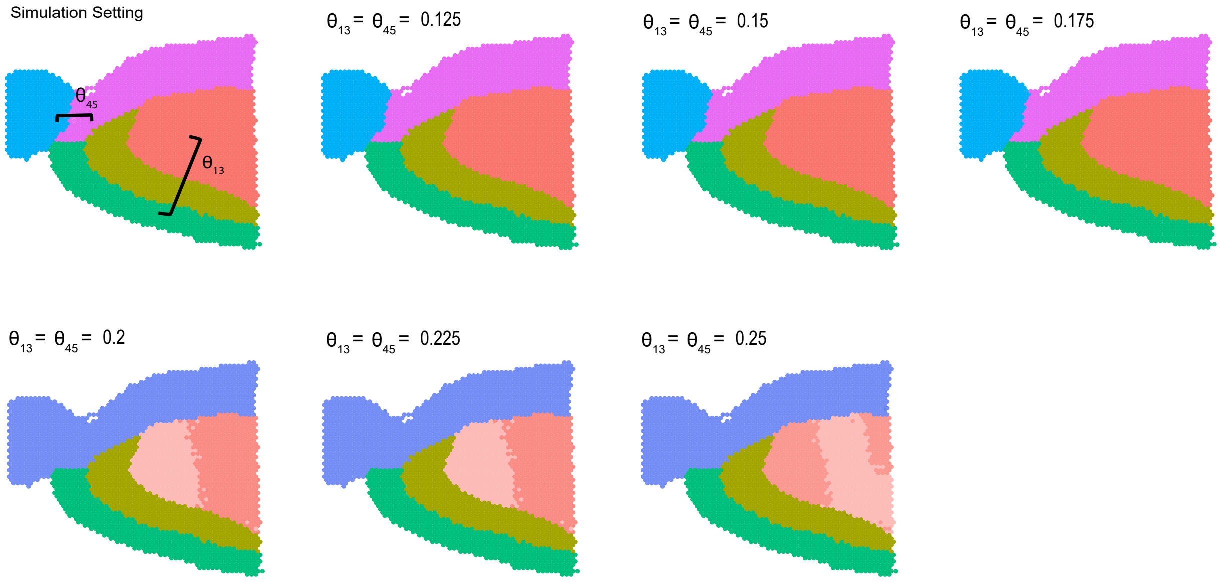

Figure S2. Simulation study: effects of between community connectivity (BCC). The number of communities is set to 5, and all the within-community connectivity (WCC) is set to 0.3. We varied the BCC between spatially close community pairs of 4 (blue) and 5 (pink) and spatially apart pairs 1 (orange) and 3 (green) from 0.125 to 0.25, while keeping all the rest BCC to be 0.1.

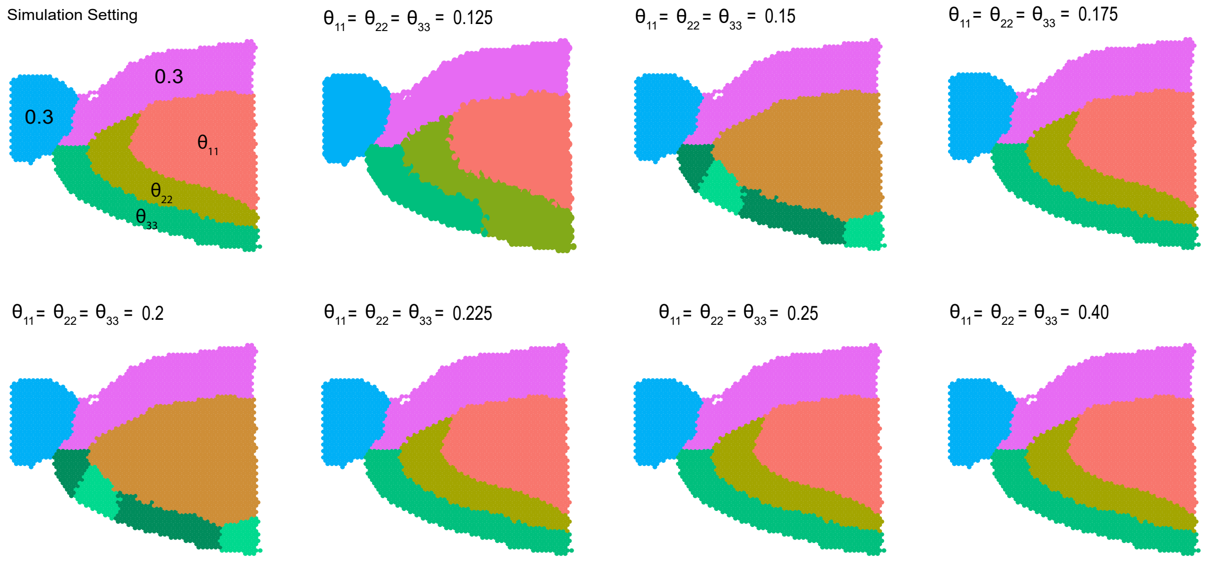

Figure S3. Simulation study: effects of within community connectivity (WCC). The number of communities is set to 5, all the between-community connectivity (BCC) is set to 0.1. We varied the WCC for community 1, 2, and 3 (orange, olive, and green, respectively) from 0.125 to 0.40, while kept all the rest WCC to be 0.3.

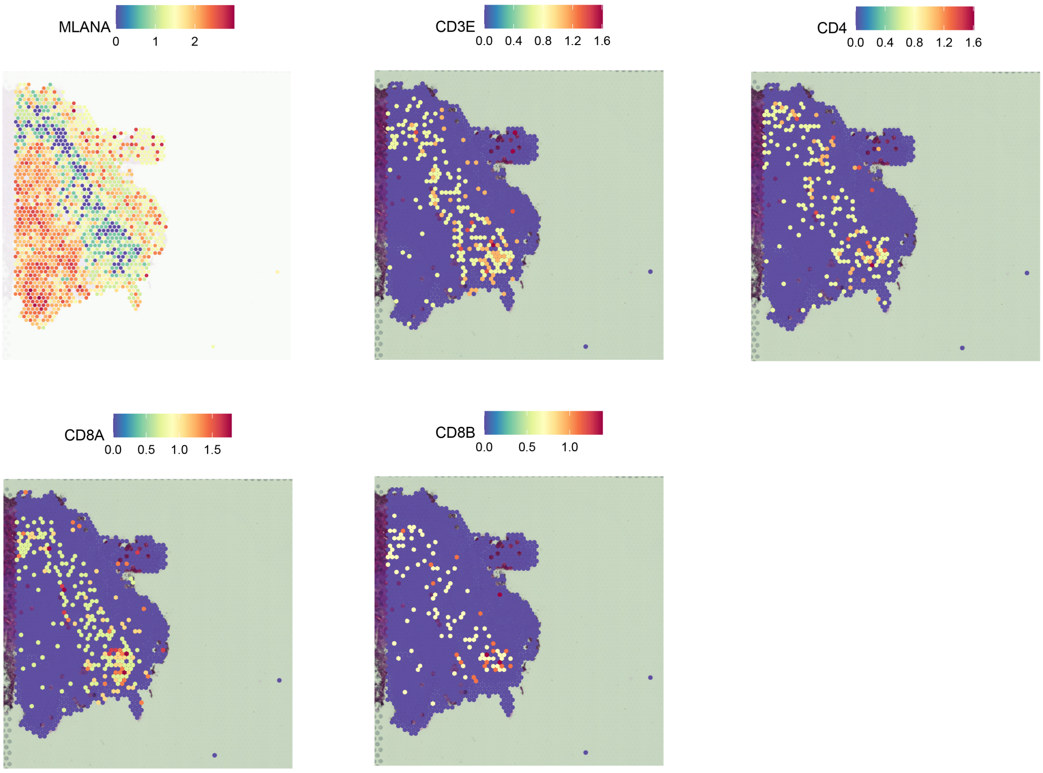

Figure S4. The spatial distribution of marker genes for the brain metastasis data.

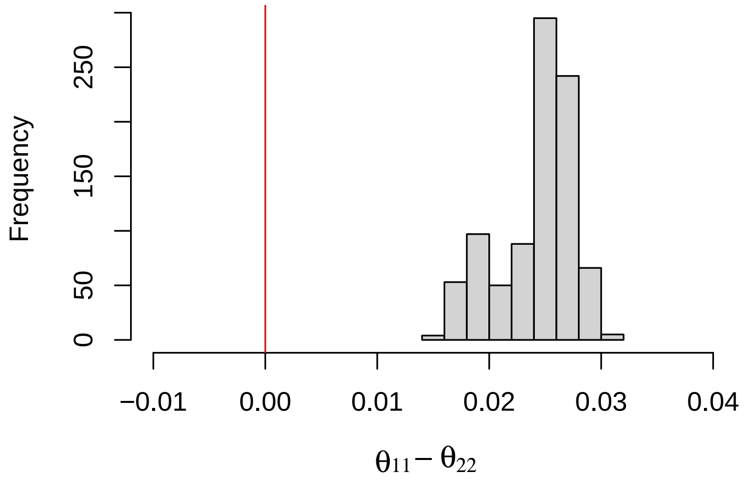

Figure S5. Histogram of the posterior distribution of $\theta_{11} - \theta_{22}$ for the brain metastasis data.

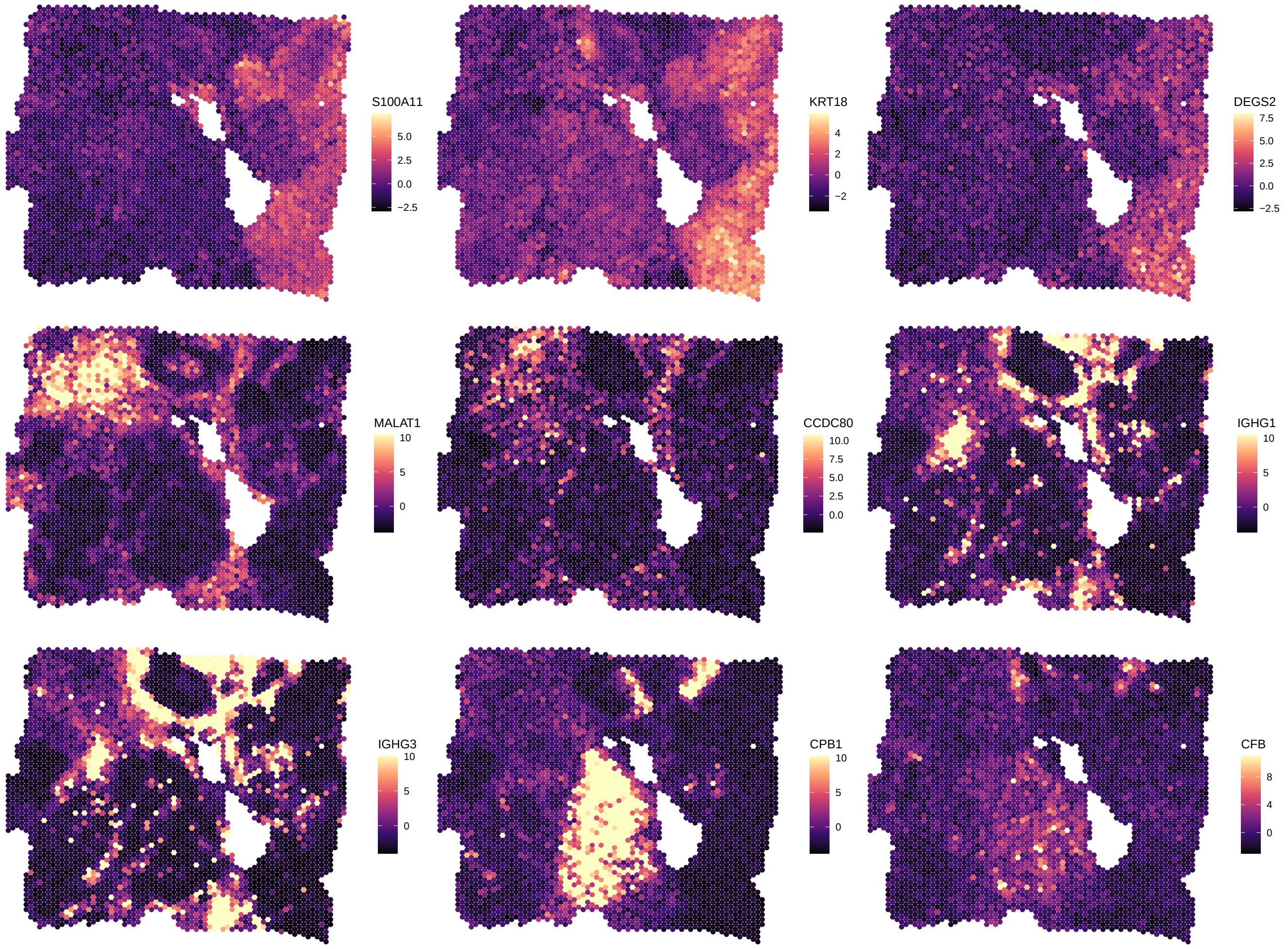

Figure S6. The spatial distribution of selected differentially expressed genes for each sub-population on the invasive ductal carcinoma tissue sample.

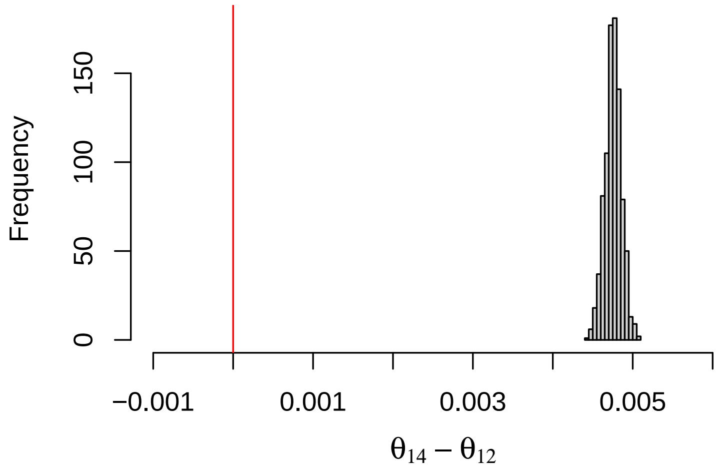

Figure S7. Histogram of the posterior distribution of $\theta_{14} - \theta_{12}$ for the invasive ductal carcinoma data.

1 T CELL REPERTOIRE (TCR) SIMILARITY AND DIVERSITY

1.1 TCR similarity

The similarity of TCR repertoire between sub-population g and k is defined as the inverse of the distance between them, i.e.,

$$\frac{1}{d_{gk}} = \frac{1}{dist(\mathbf{x_g}, \mathbf{x_k})}, \tag{S1}$$

where x_{ik} denotes the frequency of clonotype i in sub-population k, R denotes the number of unique clonotypes that appear in at least one of the sub-populations, $\mathbf{x_g} = (\frac{x_{1g}}{x_{.g}}, \frac{x_{2g}}{x_{.g}}, ..., \frac{x_{Rg}}{x_{.g}})^T$, and $\mathbf{x_k} = (\frac{x_{1k}}{x_{.k}}, \frac{x_{2k}}{x_{.k}}, ..., \frac{x_{Rk}}{x_{.k}})^T$.

1.2 TCR diversity

The diversity of the TCR repertoire for a sub-population is evaluated using the inverse Simpson's index, which is defined as follows:

$$\frac{1}{\sum_{i=1}^{R} p_i^2} \tag{S2}$$

where R denotes the number of unique clonotypes in the sub-population, and p_i denotes the proportion of clonotype i. The higher value of inverse-Simpson's index indicates the higher level of diversity.

2 WILCOXON RANK-SUM TEST

The Wilcoxon rank-sum test is a non-parametric alternative to the independent two-sample t-test. It is used for comparing two independent groups of samples without assuming the normality. Let $X_1, ..., X_{n_1}$ be i.i.d sample from group 1, and $Y_1, ..., Y_{n_2}$ be i.i.d sample from group 2. To test if the two groups are equal or not, we first pool the data from the two groups and assign ranks to each data. Then we sum the ranks in groups 1 and 2, respectively. Next, we compute the following test statistic:

$$U = min(U_1, U_2)$$

where $U_1 = n_1n_2 + \frac{n_1(n_1+1)}{2} - R_1$,$U_2 = n_1n_2 + \frac{n_2(n_2+1)}{2} - R_2$, with R_1 and R_2 being the sum of ranks for groups 1 and 2, respectively. The computed test statistic is then compared to the critical value from the Wilcoxon rank-sum distribution to determine significance.
